# Dose and Route of rBCG-LTAK63 Immunization Determine Protective Efficacy and Pathological Outcome in Tuberculosis

**DOI:** 10.3390/vaccines14100845

**Published:** 2026-09-24

**Authors:** Lázaro M. Marques-Neto, Monalisa M. Trentini, Ana Carolina R. Moreno, Luana Moraes, Silas F. Eto, Giana C. Gaboardi, Alex I. Kanno, Luciana C. C. Leite

**Affiliations:** Laboratório de Desenvolvimento de Vacinas, Instituto Butantan, São Paulo 05503-900 14, Brazil

**Keywords:** tuberculosis, recombinant BCG, dose, delivery-route, vaccine

## Abstract

Background/Objectives: BCG shows variable protection against adult pulmonary tuberculosis. Recombinant BCG platforms, such as rBCG-LTAK63, aim to improve efficacy, but how dose and route influence protection versus immunopathology remains to be determined. We investigated how these factors shape bacterial control, lung injury, inflammation, and CD4^+^ memory. Methods: We established a dose–response by subcutaneous (SC) immunization and then fixed a high dose (1 × 10^7^ CFU) to compare SC, intranasal (IN), and intravenous (IV) administration. Lung CFU, histopathology, lung cytokines, and CD4^+^ memory phenotypes in lung and spleen were quantified. Results: rBCG-LTAK63 showed a clear SC dose–response, with higher doses improving bacterial control and immunogenicity. At the fixed high dose, outcomes diverged by route. IN vaccination produced the greatest reduction in lung CFU but also induced marked lung injury, with increased inflammation, fibrosis, necrosis, together with elevated TNF, IL-6, and IL-17. On the other hand, IV delivery achieved robust bacterial control with minimal pathology and promoted IFN-γ–skewed CD4^+^ responses enriched for lung central and tissue-resident memory. SC resulted in the lowest bacterial control but still a ~2 log reduction, and predominantly systemic responses. These patterns reveal distinct correlates: IFN-γ with lung TCM/TRM associated with protection, and TNF/IL-6/IL-17 with inflammatory/TEM bias associated with pathology. Conclusions: rBCG-LTAK63 safety and efficacy are strongly route- and dose-dependent. The same high antigenic dose is beneficial via IV yet crosses an inflammatory threshold via IN. These findings support route-specific dose optimization, including defining minimal effective IV doses and maximal safe IN doses, and motivate evaluation of fractional, heterologous, or mixed-route regimens. Understanding how protection and pathology uncouple by route is critical for advancing rBCG-LTAK63 toward clinical development.

## 1. Introduction

Tuberculosis (TB) continues to be the leading cause of mortality globally from a single infectious agent [1]. The Bacille Calmette–Guerin (BCG) vaccine, developed more than a century ago, remains the only licensed vaccine against tuberculosis [2]. While it is very effective when administered to newborns, it offers limited protection against adult pulmonary tuberculosis [2]. Several strategies based on recombinant mycobacteria and recombinant proteins are under investigation with promising results, such as VPM1002 [3], MTBVAC [4], and M72F/AS01E [5,6]. Additionally, innovative delivery approaches are being explored to enhance the immunological response elicited by BCG, including BCG revaccination, alternate administration routes, and the utilization of BCG in prime-boost regimens with novel vaccine candidates.

A genetically detoxified variant of the *E. coli* heat-labile enterotoxin, LTK63, is recognized for its robust mucosal adjuvanticity [7,8]. We have previously constructed a recombinant BCG strain, designated rBCG-LTAK63, that expresses the A subunit of LTK63 as an adjuvant. Immunization of mice with rBCG-LTAK63 markedly augmented both innate and adaptive immune responses, offering superior protection against *Mycobacterium tuberculosis* (*Mtb*) challenge relative to conventional BCG immunization, culminating in an increased reduction in pulmonary bacterial burden [9]. Subsequent research has shown that rBCG-LTAK63-immunized mice had higher numbers of single and polyfunctional effector cells and central memory T-cells, providing enhanced protection for up to 180 days [10].

Although these results show promise, further improvements can still be pursued. One alternative involves modifying the immunization delivery strategy. Traditionally, BCG is administered through intradermal immunization; however, delivery directly to the lungs may improve the protective response at the primary infection site [11]. Research assessing the protective efficacy of BCG or other vaccine candidates administered by aerosol or intranasal methods has demonstrated variable performance across several animal models, including mice and non-human primates (NHPs) [12,13,14]. Several vaccine delivery modalities, including intravenous (IV) and intranasal (IN) routes, have been investigated with the objective of enhancing the systemic immune response [15,16,17]. In NHPs, high-dose intravenous BCG immunization conferred complete protection against *Mtb* challenge in most animals [18]. However, in mice, results from intravenous BCG appear inconsistent, failing to reproduce the enhanced protection relative to subcutaneous delivery that has been reported in NHPs [19].

While there are no definitive correlations of protection against *Mtb* infection, certain immune responses are crucial for disease protection. A cell-mediated T-helper 1 (Th1) response from CD4 T-cells and an MHC-I restricted CD8 T-cell response are essential for infection control and are often used to assess vaccine immunogenicity. Polyfunctional CD4 T-cells, which express cytokines such as IFN-γ, TNF-α, IL-2, or IL-17, may play a role in disease progression and indicate vaccine-induced protection [20]. Successful immunization against TB must stimulate effector T-cells (TEM) and develop T-cell memory populations for long-term protection. Although TCM populations have been identified following systemic BCG vaccination, it is believed that the current vaccine primarily elicits TEM populations that provide transient control of *Mtb* [21,22]. Enhancing TCM or tissue-resident memory (TRM) populations may provide rapid and sustained protection [23,24].

Here, we investigated the effects of different routes of administration, with higher doses of rBCG-LTAK63, on eliciting a protective immune response against active tuberculosis, leveraging the success of alternative vaccination methods such as high-dose IV BCG, which enhanced protection in NHPs.

## 2. Materials and Methods

### 2.1. Animals, Strains and Immunization

Specific-pathogen-free female BALB/c mice, aged 4 to 8 weeks, were obtained from the Instituto Butantan Central Animal Facility (São Paulo, Brazil) and accommodated in ABSL-2 racks including HEPA-filtered air intake and an exhaust system. The mice were housed at the Laboratório de Desenvolvimento de Vacinas animal care facility with unrestricted access to water and food. Environmental parameters were regulated, maintaining a temperature range of 20–24 °C, relative humidity of 40–70%, and a 12 h light/dark cycle. This study was executed in strict compliance with the Guide for the Care and Use of Laboratory Animals, adhering to the recommendations of the Sociedade Brasileira de Ciência em Animais de Laboratório (SBCAL), and received approval from the Animal Research Ethical Committee of Instituto Butantan (protocol number: 3435250619).

The rBCG-LTAK63 strain utilized in this research has been previously described [25]. BCG Danish and rBCG-LTAK63 cultures were cultivated in Middlebrook 7H9 medium (Difco, Detroit, MI, USA) with 10% OADC (oleic acid–albumin–dextrose–catalase; BBL, Cockeysville, MD, USA), 0.5% glycerol, and 0.05% Tween 80 (7H9-OADC), or on Middlebrook 7H10 agar supplemented with 0.5% glycerol and OADC (7H10-OADC).

To evaluate immune responses and protection, groups of mice (*n* = 6) were immunized subcutaneously with wild-type BCG (wtBCG) or rBCG-LTAK63 (1 × 10^6^ CFU/100 μL, designated SC-10^6^) in the dorsal region, or with rBCG-LTAK63 (1 × 10^7^ CFU/100 μL) administered subcutaneously (SC-10^7^), intranasally (IN-10^7^), or intravenously (IV-10^7^) via the tail vein. All vaccine preparations were resuspended in phosphate-buffered saline (PBS; 137 mM NaCl, 2.7 mM KCl, 8 mM Na_2_HPO_4_, and 2 mM KH_2_PO_4_).

### 2.2. Mycobacterium tuberculosis H37Rv Challenge Experiments

A frozen vial of *Mycobacterium tuberculosis* H37Rv, maintained at −80 °C, was thawed, and the inoculum was prepared by diluting to its final concentration for the challenge.

Two challenge models were employed to access protective response in this work. The surgical access and intratracheal administration of 10^5^ CFU introduces the inoculum directly into the trachea, circumventing the upper airway and ensuring consistent, reproducible deep-lung deposition with precise dosage control, resulting in a severe, swiftly advancing infection ideal for assessing protection under significant bacterial load within a brief experimental timeframe. We employed intranasal administration of 500 CFU in 40 µL under anesthesia as an adjunctive non-surgical approach. At this volume, a significant fraction of the inoculum reaches the lower respiratory tract after traversing the upper airway [26]. The intranasal model facilitated a reduction of over 200-fold in the challenge inoculum, which, while still exceeding the 50–100 CFU characteristic of low-dose aerosol systems, approximates physiological exposure and leads to slower disease progression, thereby enhancing the detection of variations in sustained bacterial control. The two models were selected to examine the effectiveness of immunization across varying deposition patterns and bacterial loads [10,27,28].

In the initial experiment, animals were challenged 90 days after immunization via the intratracheal route with *Mycobacterium tuberculosis* H37Rv (10^5^ CFU/100 μL), as previously established in our laboratory. Briefly, BALB/c mice were anesthetized, and a small incision was made in the throat. Using an insulin syringe, 10^5^ viable bacteria diluted in 100 μL of PBS were injected directly into the trachea. Following the procedure, mice were maintained in a vertical position until anesthesia wore off [9,29].

In the further experiments, the intranasal infection was performed as described by Logan et al. (2008). Briefly, at 90 days following immunization, mice were intranasally challenged with 500 CFU of the *M. tuberculosis* suspension in a 40 µL volume, delivered into one nostril [27].

To confirm pulmonary delivery of the administered inoculum, one mouse per experimental group was euthanized 24 h post-infection, and lung homogenates were plated on 7H10-OADC as a technical control of the challenge procedure. The animals’ lungs were collected 30 days post-infection to evaluate protective effectiveness. The anterior and mediastinal lobes of the right lung were harvested, homogenized, and inoculated onto 7H10-OADC agar. Bacterial burden was quantified by enumerating CFU following 14–21 days of incubation at 37 °C, 5% CO_2_.

### 2.3. Cellular Immune Responses in Spleen and Lungs

Flow cytometry analysis of specific effector T-cells and memory T-cells was performed in accordance with previously described techniques [10,30]. In summary, 90 days following immunization, the spleen and lungs were collected. Spleens were processed into single-cell suspensions utilizing 70 µm cell strainers (BD Biosciences, San Jose, CA, USA), and the cells were resuspended in RPMI-1640 media enriched with 10% fetal calf serum, 0.15% sodium bicarbonate, 1% L-glutamine, and 1% nonessential amino acids.

Lungs were enzymatically digested using DNase IV (30 µg/mL) and collagenase III (0.7 mg/mL) for 30 min at 37 °C. The processed lungs were converted into single-cell suspensions utilizing 70 µm cell strainers, and erythrocytes were lysed with an RBC lysis solution (0.15 M NH_4_Cl, 10 mM KHCO_3_). Viable cells from both tissues were enumerated using a Neubauer chamber with 0.2% Trypan Blue, and the cell concentration was standardized to 1 × 10^6^ cells/mL. All reagents were procured from Sigma-Aldrich^®^, Merck KGaA, St. Louis, MO, USA.

Subsequently, 10^6^ cells per well were seeded in 96-well plates (CellWells™, Sigma-Aldrich^®^, St. Louis, USA) and stimulated with 10 µg of BCG CFP (culture filtrate protein, obtained from BCG cultured in Sauton media for 14 days and concentrated using a 5000 MWCO filter) or remained unstimulated. The cultures were incubated at 37 °C with 5% CO_2_ for 4 h, followed by the addition of monensin (3 µM; eBioscience, San Diego, CA, USA), and incubation was extended for another 4 h. Cells were then exposed to 0.1% sodium azide (Sigma-Aldrich, St. Louis, USA) in PBS for 30 min at room temperature, followed by centrifugation at 400× *g* for 15 min. Cellular phenotyping was conducted through permeabilization using Perm Fix/Perm Wash (BD Pharmingen, San Jose, CA, USA) and subsequent staining with the following conjugated antibodies: TNF-α-FITC (clone MP6-XT22), IFN-γ-PE (clone XMG1.2), CD4-PerCP (clone RM4-5), CD8-PEcy7 (clone 53.6.7), CD44-APC (clone IM7), IL-17-BV421 (clone TC11-18H10), CD62L-PE (clone MEL-14), PD-1-PE (clone J43), KLRG1-APC (clone 2F1), and CXCR3-BV421 (clone CXCR3/173).

A total of 200,000 events for the spleen or lungs per sample was acquired utilizing a BD FACS Canto II flow cytometer, with data analysis performed using FlowJo™ v10 Software (BD Life Sciences, San Jose, CA, USA). The gating strategies are described in Section 2.4 and shown in Supplementary Figures S1–S5.

### 2.4. Cytometric Bead Array

Culture supernatants were harvested and cryopreserved until analysis. Cytokine quantification was performed using multiplexed Cytometric Beads Array (CBA mouse Th1/Th2/Th17 kit, BD Biosciences) to measure IFN-γ, TNF-α, IL-6, IL-17, IL-4, and IL-10. Detection limits ranged from 2.5 to 17.5 pg/mL with a maximum of 5000 pg/mL. Data was acquired on a FACS Canto II instrument and analyzed using FCAP Array Software (v3.0, BD Biosciences, San Jose, USA).

The heatmaps depict relative cytokine modulation expressed as a Z-score normalized to the saline control for each analyte and compartment. Z-scores were calculated as: Z = (value − mean_Saline)/SD_Saline, where value is the cytokine concentration for each animal. Thus, a Z-score reflects how many standard deviations an individual measurement deviates from the saline mean. Positive Z-scores (red) indicate concentrations higher than the saline mean, whereas negative Z-scores (blue) indicate concentrations lower than the saline mean; values near zero (neutral colors) indicate levels close to the saline average. Numbers shown in the center of each cell correspond to the group median Z-score (i.e., the median standardized deviation relative to saline).

### 2.5. Lung Histopathological Process and Analysis

Histopathological analysis of lung tissue was conducted to quantify inflammatory processes, pathological alterations associated with tuberculosis infection, using a previously validated scoring system. The intensity of inflammation was assessed on a scale from 0 to 4, where higher scores indicate more extensive and severe inflammatory infiltrates. The extent of necrosis was measured based on the proportion of affected tissue, ranging from 0, for the absence of necrosis, to 4, for more than 75% of the tissue being compromised. Fibrosis, considered a marker of tissue repair and healing, was evaluated with scores ranging from 1, for mild fibrosis, to 4, for extensive and dense fibrosis [31,32,33].

### 2.6. Statistical Analysis

Data analysis was conducted utilizing GraphPad Prism 9 (GraphPad, La Jolla, CA, USA). Group differences were assessed using one-way ANOVA, with *p* values < 0.05 deemed statistically significant. All biological tests were performed a minimum of two times, encompassing repeated vaccines and subsequent evaluations of immune response and protection.

## 3. Results

### 3.1. Subcutaneous rBCG-LTAK63 Induces Dose–Response Efficacy Against Mtb Intratracheal Challenge

Subcutaneous immunization with 10^6^ CFU is the established benchmark for evaluating BCG-derived vaccine candidates; however, the evaluation of different doses is important to understanding the potency of the vaccines. We performed two independent studies to assess the dose-effect of rBCG-LTAK63, using the intratracheal (IT) and intranasal (IN) challenge models. In the first experiment, the IT infection (10^5^ CFU) resulted in a high bacterial load with ~4 × 10^7^ CFU/mL (Figure 1A). Interestingly, the challenge with 500 CFU in the intranasal (IN) model (Figure 1B) reached a comparable baseline infection of ~2 × 10^7^ CFU/mL. Although the IN route is considered to be more physiological, the median bacterial recovery showed a marginal difference between the two models. This indicates that both methods are technically equivalent in supporting robust *Mtb* expansion, providing a consistent baseline for efficacy testing.

In both IT and IN challenge models, rBCG-LTAK63 demonstrated enhanced pulmonary protection as compared to conventional BCG (at 10^6^ and 10^7^ CFU). Initial assessment using a dose-escalation strategy (10^4^ to 10^7^ CFU) followed by IT challenge revealed that protection is strictly sensitive to the vaccine inoculum, with the 10^7^ CFU dose achieving the most robust reduction in lung bacterial burden (Figure 1A). This protective effect was reproduced in the IN-challenge model (Figure 1B–H), where rBCG-LTAK63 outperformed standard BCG, consistently yielding the lowest median bacterial recovery.

Furthermore, a dose of 10^5^ CFU of rBCG-LTAK63 is equivalent to the 10^6^ CFU of BCG. The dose response in protective efficiency was evident, with the 10^7^ CFU rBCG-LTAK63 dose showing the highest protection. The dose also correlated with the preservation of pulmonary architecture. Histopathological analysis showed that both BCG and rBCG-LTAK63 mice receiving the 10^6^ CFU dose exhibited significantly lower scores for inflammation (Figure 1C,F) and necrosis (Figure 1D,F). On the other hand, the fibrosis scores were only reduced in the groups receiving rBCG-LTAK63; the group receiving rBCG-LTAK63 at 10^6^ CFU showed the lowest pathology (Figure 1F). Although rBCG-LTAK63 immunization with 10^7^ CFU induced increased protection, these animals exhibited a trend towards higher inflammation scores (Figure 1C), consistent with the histological sections showing a larger proportion of tissue lesion (Figure 1F).

The superior protection was associated with a distinct immunomodulatory signature characterized by the potentiation of the Th1/Th17 axis. Ex vivo cytokine profiling of lung culture supernatants (Figure 1G) revealed that immunized groups tend to display cytokine levels lower than the non-immunized infection control in the lungs. Among the groups immunized with 10^6^ CFU, rBCG-LTAK63 induced a more robust Th1 profile in the lungs as compared to conventional BCG, with higher scores for IFN-γ, TNF-α, while IL-17 was decreased. On the other hand, when the dose was increased to 10^7^ there was a strong increase in IL-17 while IFN-γ decreased. This pattern indicates that rBCG-LTAK63 modulates the quality of the pulmonary response in a dose-dependent manner. In the spleen, at higher doses there is a shift towards a stronger Th1/Th17 profile, indicating a more robust systemic response (Figure 1H).

### 3.2. High Dose Immunization Potentiates Th1/Th17 Immune Responses Before Challenge

Since rBCG-LTAK63 demonstrated dose-dependent protection, we investigated whether higher doses administered via the subcutaneous route could improve the immunological readouts prior to challenge. Immunological profiles were evaluated 90 days post-immunization to characterize the baseline immunogenicity of effector cells right before the infection. Regarding the recruitment of antigen-specific cells to the lung parenchyma, rBCG-LTAK63 immunization (10^6^ and 10^7^ CFU) significantly increased the frequency of IFN-γ, TNF-α, and IL-17–producing CD4^+^ T-cells compared to the saline control and to the standard BCG dose (Figure 2A). Additionally, the dose increase improved the pulmonary immune response (IFN-γ, TNF-α, and IL-17–producing CD4^+^ T-cells) establishing a robust density of effector cells. In the spleen, rBCG-LTAK63 elicited Th1-predominant responses mostly by IFN-γ and TNF-α production higher than control saline or BCG, with rBCG-LTAK63 also displaying a dose response (Figure 2B).

Polyfunctional cells analysis in the lungs revealed that both BCG and rBCG-LTAK63 expanded specific CD4^+^ T-cell subsets, showing higher proportions of double-positive (IFN-γ^+^/TNF-α^+^, IFN-γ^+^/^+^IL-17^+^, and TNF-α^+^/^+^IL-17^+^) and triple-positive (IFN-γ^+^/TNF-α/^+^IL-17^+^) cells (Figure 2C), with the high dose displaying the highest percentages. The dose response effects equally all the polyfunctional populations. In the spleen, the double functional IFN-γ^+^/TNF-α^+^ cells represent the major polyfunctional population and were further increased in the high dose group, whereas other multifunctional subsets contributed minimally (Supplementary Figure S3B). Collectively, these data indicate that rBCG-LTAK63 enhances protection by inducing a potent cellular response, prioritizing the expansion of Th1 and Th17 in the lungs, but mainly systemic Th1 single and polyfunctional effector cells, that are ready for rapid mobilization upon *Mtb* encounter.

### 3.3. Dose of Immunization Improves Generation of TCM but Not TRM in the Lungs

Previous work showed that subcutaneous immunization with 10^6^ CFU of rBCG-LTAK63 increases the CD4^+^ T-cell memory landscape in BALB/c mice up to 180 days post-immunization, establishing a sustained state of immunological readiness prior to challenge [10]. Building on these findings, we next evaluated how a high-dose formulation of rBCG-LTAK63 (10^7^ CFU) influences the development and magnitude of memory T-cell responses. Increasing the dose led to a pronounced increase in naïve, TCM and TEM CD4^+^ T-cell population in the lungs (Figure 3A–C). In contrast, in the spleen, naïve and TCM CD4^+^ T-cells decreased at 10^7^ CFU relative to the 10^6^ CFU group, contrary to that observed in the lungs. This divergence suggests that higher antigen load preferentially drives a shift toward effector memory in systemic compartments while still supporting TCM maintenance or expansion within the lung microenvironment.

In contrast, lung tissue-resident memory cells (TRM) displayed a qualitatively distinct pattern (Figure 3D). Previous study shows a tendency to increase in this population [10], and now the refined panel incorporating the CXCR3 marker confirmed that subcutaneous immunization with rBCG-LTAK63, but not parental BCG, effectively generated CD4^+^ TRM in the lung parenchyma. Notably, TRM frequencies did not increase further with the high dose, suggesting that the threshold required for residency establishment is already achieved at 10^6^ CFU, whereas amplification of systemic memory subsets requires higher antigenic load. The data in Figure 3A–G indicate that rBCG-LTAK63 promotes a dual protective architecture, with a dose-dependent expansion of systemic memory pools (TEM/TCM) and a dose-independent establishment of lung-resident sentinels, which likely enhances vaccine efficacy by combining rapid local responsiveness with sustained systemic recall.

### 3.4. Route of Administration Dictates the Protective Efficacy and Pathological Outcomes of High-Dose rBCG-LTAK63 Immunization

Building on the work of Darrah et al. [18] and our prior observation that increasing the rBCG-LTAK63 dose improves outcomes, we fixed a high dose (10^7^ CFU) and compared routes of administration to determine route-dependent effects on protection. Among the tested routes, intranasal (IN) immunization produced the strongest reduction in lung bacterial burden, achieving ~4-log decrease compared with saline controls. Intravenous (IV) vaccination conferred an average 3-log reduction, whereas subcutaneous (SC) administration confirmed the previous results with ~2-log protection (Figure 4A). However, bacterial control did not translate into proportional tissue preservation. Despite superior CFU reduction, the IN route showed worse lung pathology, with elevated scores for inflammation, fibrosis and necrosis (Figure 4B–D), comparable to the infected only group. Histopathology corroborated this pattern: IN-vaccinated lungs displayed disrupted architecture and large inflammatory foci, whereas IV vaccination preserved tissue structure more effectively, and SC exhibited milder, more localized lesions (Figure 4E). Thus, at this dose, IN delivery enhances bacterial clearance but simultaneously exacerbates lung damage, uncoupling pathogen control from tissue protection.

Cytokine profiles mirrored these compartment-specific outcomes. In the lung, intranasal (IN) vaccination generated the strongest pro-inflammatory response relative to infected controls and to the other routes, with marked increases in TNF-α, IL-6, IL-17, and IL-10 (Figure 4F). This exaggerated inflammatory signature aligns with the increased pathology observed in IN-vaccinated animals. In contrast, intravenous (IV) immunization produced a cytokine pattern characterized by better controlled inflammatory mediators (TNF-α and IL-17), consistent with better tissue preservation and controlled inflammation. Subcutaneous (SC) delivery induced a lower cytokine response as compared to infected controls but a modest increase in IL-4.

In the spleen, IN vaccination again increased inflammatory cytokines, particularly TNF-α and IL-17 (Figure 4G). IV vaccination increased TNF-α and IL-6 systemically relative to controls, while SC produced a strong increase in IFN-γ production, but less than IN, and a decrease in IL-6 production (Figure 4G). Together, these data show that at a high dose, the IV route achieves the most balanced response, improving bacterial control while maintaining cytokine levels. In contrast, the IN route, although superior in reducing bacterial burden, drives an inflammatory response that exceeds both infected controls and other vaccinated groups, revealing a route-specific liability in tissue preservation.

### 3.5. Route-Dependent CD4^+^ T-Cell Responses in the Lungs and Spleen

To determine how the route of administration shapes the immune response at a fixed high dose of rBCG-LTAK63, we quantified pre-challenge CD4^+^ cytokine-producing T-cells 90 days after immunization. In the lung, IV delivery consistently generated the highest frequencies of CD3^+^CD4^+^ T-cells producing IFN-γ (Figure 5A), TNF-α (Figure 5B), and IL-17 (Figure 5C), indicating superior local Th1 and Th17 imprinting compared with SC or IN routes. Concordantly, IV vaccination produced the largest proportion of dual- and triple-cytokine-positive CD4^+^ T-cells in the lung (Figure 5D), demonstrating enhanced polyfunctionality at the site of infection.

The systemic responses showed the same hierarchy, with IV rBCG-LTAK63 (10^7^ CFU) inducing the highest frequencies of CD3^+^CD4^+^ IFN-γand TNF-α^+^ (Figure 5B) T-cells, indicating stronger systemic Th1 priming than IN or SC routes. Splenic CD4^+^IL-17^+^ T-cells remained uniformly low across routes, consistent with preferential Th17 localization to mucosal compartments. Interestingly, the polyfunctional splenic CD4^+^ responses were more pronounced following SC administration for IFN-γ^+^TNF-α^+^, while IN induced greater frequency of IFN-γ^+^IL-17^+^ double-positive subsets (Figure 5D). This data indicates that although IV is better to prime Th1/Th17 response the approach generated primary single producing cytokine cells.

To extend the route-specific analysis of high-dose rBCG-LTAK63, we quantified CD4 memory subsets in the lungs and spleen. In the lungs, naïve frequencies were lowest after IN delivery, reduced compared to saline, SC, and IV (Figure 6A). TCM increased in all vaccinated groups compared with saline, with IV showing the highest frequency, followed by IN and SC respectively (Figure 6B). In contrast, IN produced the highest TEM frequencies, exceeding SC and IV, and all three vaccinated groups displayed higher TEM levels than the saline baseline (Figure 6C). TRM also showed a stepwise increase over saline across all rBCG routes, with IV inducing the highest levels, followed by IN and SC (Figure 6D).

In the spleen, all vaccination routes decreased naïve CD4 levels relative to saline, and among the immunized groups, IN maintained the highest naïve frequencies, followed by IV and SC showing the more prominent reduction (Figure 6E). TCM was induced by IV and IN immunization, with IV eliciting the strongest response and IN a less prominent increase, whereas SC did not enhance TCM relative to the control (Figure 6F). TEM increased after all vaccination routes; however, IV generated the highest levels of this cell population, surpassing SC and IN, which remained similar to each other (Figure 6G). Overall, each delivery route imprints a distinct CD4 memory profile. IV maintains naïve levels in the lung while strongly increasing TCM and TRM, and in the spleen it enhances both TCM and TEM. IN reduces naïve frequencies in the lung and is the most effective route for expanding lung TEM, while maintaining the highest splenic naïve levels among vaccinated groups. SC consistently induces lower responses across compartments, generating weaker memory differentiation relative to IV and IN.

## 4. Discussion

Previous studies using rBCG-LTAK63, using a subcutaneous dose of 10^6^ CFU, demonstrated that the expression of the adjuvant exerts its effect mainly by strengthening innate activation and driving better long-term T-cell memory; this translates into improved protection against *Mtb* in mice. In the above studies, rBCG-LTAK63 increased early neutrophil/macrophage recruitment, nitric oxide and hydrogen peroxide production, CD4+ migration [29], and long-term TCM and TRM, as well as the Th1/Th17 single and multifunctional profile [10]. This mechanism was further investigated, linking rBCG-LTAK63-immunization to stronger inflammasome-associated IL-1β production, that can enhance CD4+ T-cell activation, Th1/Th17 polarization, and protection in mice, as compared to parental BCG [34]. Systems-biology analysis also connected the expression of LTAK63 to hypoxia, autophagy and circadian gene regulation, which correlated with improved immune response, reduced bacterial burden, and lower lung pathology [28].

Here we evaluated if different dose and routes could improve the effect of rBCG-LTAK63. Our data show that both route and dose of rBCG-LTAK63 deterministically shape the balance between protective immunity and immunopathology. The dose–response experiments show a clear SC dose–response foundation, with graded improvements in protection and immunogenicity supporting selection of a fixed high dose (10^7^ CFU) for subsequent route comparisons (Figure 1, Figure 2, Figure 3 and Figure 4). At this dose, we observe a striking dichotomy: high-dose IN vaccination achieves the greatest reduction in pulmonary bacterial burden but at the cost of exacerbated lung pathology, whereas IV vaccination provides robust protection with substantially less tissue damage. SC consistently performs weaker, producing predominantly systemic imprinting.

Routes of administration drive distinct yields of T-cell cytokine milieus that align with the observed outcomes. IN vaccination elicits strong local and systemic Th1/Th17 responses (IFN-γ and IL-17) and leads to marked elevation of TNF-α, IL-6, IL-17, and IL-10 in the lung after challenge. These inflammatory signatures coincide with preferential expansion of lung TEM upon immunization. While IL-17 can facilitate early *Mtb* control via neutrophil recruitment, excessive neutrophil-driven inflammation and sustained pro-inflammatory cytokines promote collateral tissue damage and necrotic granulomas [13,14,15]. Consistent with reports that mucosal vaccination can establish strong lung TRM, our findings draw attention to the fact that overly robust or insufficiently regulated TEM/TRM responses may precipitate immunopathology upon antigen re-encounter [24,35,36]. In contrast, IV vaccination drives a more balanced Th1/TCM-dominant program, enriching lung TCM and TRM and generating an IFN-γ-skewed recall response that supports better granuloma organization and bacterial control. Although IN surpasses IV in reducing lung CFU at this high dose, IV induces the most comprehensive memory response across compartments, while IN’s dominance is largely confined to lung TEM.

Our results support a non-linear, compartmentalized dose–response, where high-dose IV delivery enhances high-quality lung memory, with single and polyfunctional cells without inducing excessive lung inflammation. The same antigenic mass delivered through the airway mucosa drives mainly TEM but after challenge amplifies TNF-α in the lungs and spleen, as well as IL-17 in spleen; this overproduction can be the responsible for worsening the pathology. These patterns align with prior work showing that high-dose IV BCG produces superior protection and extensive lung homing of antigen-specific T, whereas mucosal vaccination enhances local immunity but risks immunopathology at higher doses or with certain formulations [14,15,16,27]. The implication is that each route has a distinct therapeutic window; optimization must aim to maximize lung TRM and IFN-γ while staying below inflammatory inflection points such as IL-6 and late IL-17.

Positioning our findings alongside Darrah et al. 2020, the IV responses are congruent with the paradigm that high-dose IV BCG can approach near-sterilizing immunity by enabling widespread antigen presentation and seeding durable TCM that rapidly differentiate into IFN-γ–producing effectors upon challenge [18,19,37,38]. Fewer studies directly compare IV and mucosal routes head-to-head; in NHPs IV consistently outperforms other routes, whereas murine results are more variable and context-dependent [19,37]. Using a recombinant BCG within a single experimental framework, we demonstrate how route determines both antibacterial efficacy and tissue costs: high-dose IN translates into superior bacterial reduction with substantially worse lung injury, whereas IV achieves strong protection with comparatively lower pathology.

Translationally, these data reinforce the need for route-specific dose optimization. For IV delivery, practical and safety consideration, such as transient bacteremia and administration logistic, requires identification of the minimal effective dose that preserves lung control and TCM/TRM enrichment through step-down titration or fractional dosing [13,18].

A protective vaccine should reduce infection associated immunopathology as compared to the infected control. Hence, intravenous and subcutaneous immunization consistently reduced semiquantitative scores for inflammation, fibrosis and necrosis, whereas scores in the intranasal group were not reduced relative to non-immunized infected controls. This separation also explains why the difference in bacterial load between IN and IV should not be seen as clinical superiority since the route with the largest drop in CFU is the one with no histological improvement. In the lack of a demonstrated correlate of protection, and because tuberculosis is a disease of immunopathology as much as bacterial reproduction, we consider the tissue pathology outcome the more relevant discriminator between methods. Our data does not support 10^7^ CFU as a favorable dose for IN administration since it did not diminish post-challenge pathology. We note that lung histopathology was not assessed pre-challenge; therefore, the contribution of immunization to the post-challenged pathology cannot asserted. Dose de-escalation could maintain protection with lower tissue damage.

For SC delivery, a regulated antigen release may improve pulmonary efficacy without increasing inflammatory load. Conceptually, a multi-objective optimization framework, maximizing the decrease in CFU while restricting histopathology and inflammatory indicators like TNF-α, IL-6, and IL-17, could enable reasonable tuning and avoid trading protection for damage.

BCG, depending on route and dose, causes a persistent infection in mice. At this stage antigen is still available, and the effector pool has not fully contracted; the readout therefore reflects a mixture of residual effector activity, innate/trained mechanisms and incompletely differentiated memory rather than a true recall response. Therefore, the 90-day challenge aims to recall the specific T-cell response elicited by BCG [39,40]. In contrast, a 180-day interval may lead to a fading of response. By 90 days (≈12–13 weeks) the effector-to-memory transition is finished, the memory compartment is stable, and germinal center-dependent affinity maturation has had adequate time to occur [41,42]. In our prior characterization of this platform, this compartment was defined at 90 days and sustained through 180 days; in this case challenge elicited the anticipated recall signature [10,28]. This is also a common approach in the murine BCG/*Mtb* literature, where a ~12-week rest period is used for this exact reason [40,43].

LTAK63 is a genetically detoxified derivative of the A subunit of *E. coli* heat-labile enterotoxin. Although this specific construct has been documented essentially in the tuberculosis/BCG context, the broader family of detoxified LT derivatives has been investigated across vaccine platforms: LTK63 improved mucosal IgA responses with influenza vaccine in adults [44,45], enhanced antibody and bactericidal responses with a meningococcal C conjugate in mice [46] and was used with Ag85B-ESAT-6 to boost prior BCG immunity [47]. However, trials of LTK63 as a free intranasal adjuvant were interrupted after cases of transient Bell’s palsy, attributed to ganglioside binding by the B subunit and retrograde olfactory transport of soluble toxin [48]. This precedent is directly relevant here as we evaluate a mucosal route. In rBCG-LTAK63; however, the derivative is expressed intracellularly by a live attenuated vector rather than instilled as free protein, and only the A subunit is present. Whether this dissociates the construct from that risk remains untested, reinforcing the need for dedicated safety assessment of the intranasal route.

As limitations of this study is important to point out that the findings derive from a murine model using a single mouse strain (BALB/c) and a single sex (female), which precludes assessment of strain- and sex-dependent variation in vaccine responses and limits generalizability. Challenge was performed with the laboratory reference strain H37Rv at fixed time points and therefore does not address the diversity of clinical isolates, including drug-resistant and hypervirulent lineages. Some of the experiments did not include parallel parental BCG or empty rBCG vector controls, so the independent contribution of the LTAK63 adjuvant cannot be formally isolated from effects attributable to the BCG backbone or to recombinant vector expression itself. Protection was evaluated at a defined interval; long-term durability, both at later post-immunization intervals and with extended post-challenge follow-up, was not assessed in this cohort. Pulmonary histopathology was not obtained prior to challenge, so vaccine-associated lung pathology cannot be excluded, particularly in the higher-dose intranasal groups, and additional safety readouts, including weight dynamics, clinical scoring and extrapulmonary dissemination, were not systematically collected. Finally, cytokine–pathology associations reported here remain correlative; causal attribution will require perturbation approaches such as cytokine blockade or genetic models.

Addressing these limitations defines the priorities for further development of this platform: route-specific dose–response studies incorporating histopathological and toxicity endpoints to identify protective yet non-pathogenic operating windows; inclusion of parental BCG and vector controls to resolve the specific contribution of LTAK63; evaluation across sexes, mouse strains and clinically relevant *Mtb* isolates; extended durability studies; exploration of heterologous or mixed-route regimens to consolidate lung TRM with controlled inflammation; and multivariate modeling to define immune correlates of protection versus pathology beyond univariate trends.

## 5. Conclusions

rBCG-LTAK63 improves selected attributes of BCG, but its overall efficacy–safety profile is strongly shaped by a dose–route interaction. Increasing dose enhanced protection, and at high dose the outcome became route-dependent: intranasal delivery produced the largest reduction in pulmonary bacterial burden but failed to confer the histopathological benefit observed with the intravenous and subcutaneous routes, with scores trending above those of unvaccinated infected controls. Defining this protection–pathology balance is essential for rational vaccine design and supports advancing rBCG-LTAK63 towards clinical evaluation. The route is not a delivery detail, it is a biological intervention that reshapes risk, benefit, and the translational trajectory of the vaccine.

## Figures and Tables

**Figure 1:**
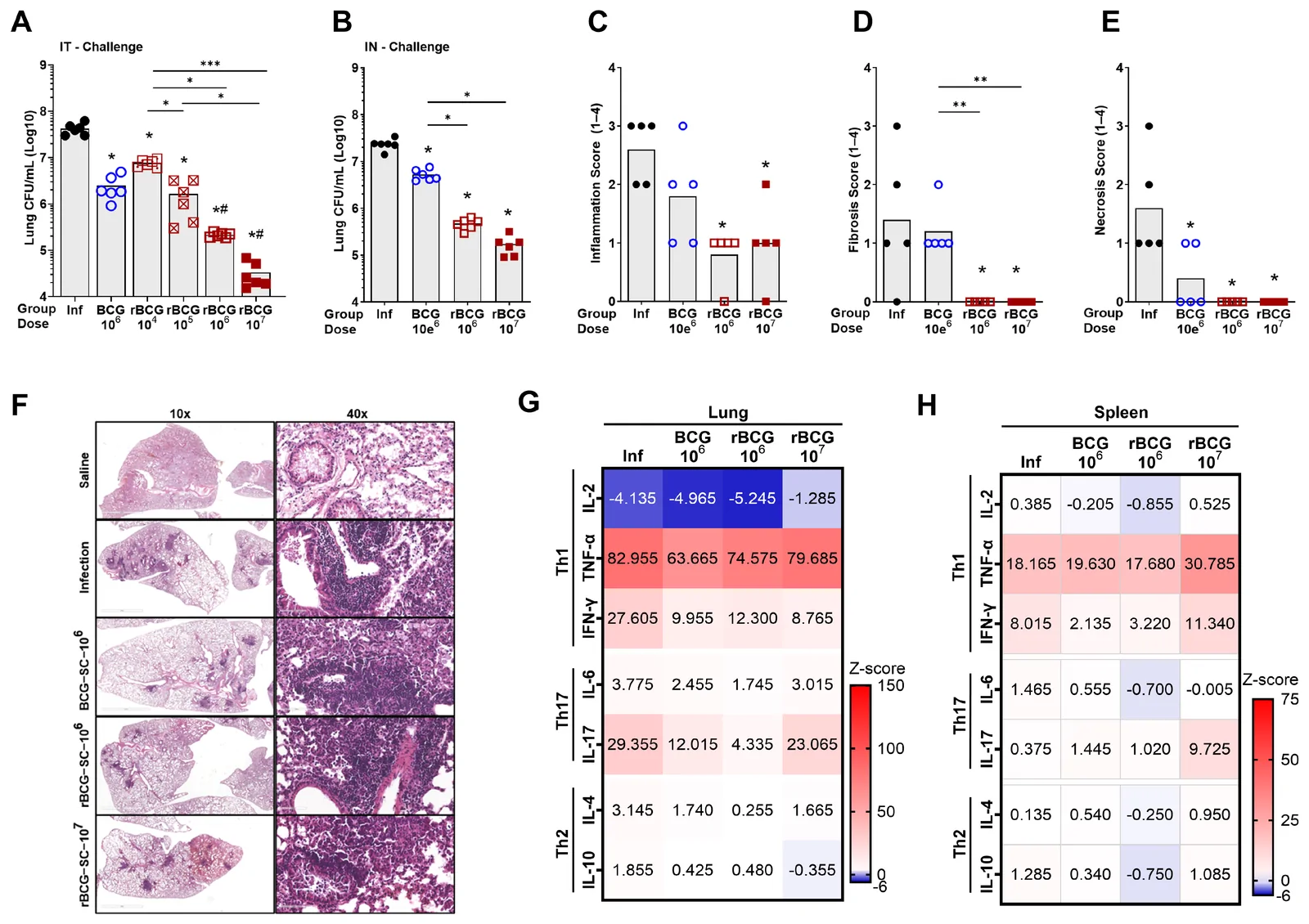
rBCG-LTAK63 vaccination confers dose-dependent lung protection and modulates inflammatory profile following *M. tuberculosis* challenge. Pulmonary bacterial burden (log_10_ CFU/mL) in mice receiving a dose-escalation immunization of rBCG-LTK63 (10^4^ to 10^7^ CFU) via the subcutaneous (SC) route. After 90 d, animals were challenged (**A**) intratracheally with 10^5^ CFU of *Mtb* H37Rv, or (**B**) an intranasal challenge with 500 CFU of *Mtb* H37Rv in 40 µL. Assessment of CFU was performed 30 days post-infection. Groups included saline (non-infected control), infection (infected non-immunized control), BCG (1 × 10^6^ CFU), and rBCG-LTAK63 (1 × 10^4^, 1 × 10^5^, 1 × 10^6^ or 1 × 10^7^ CFU). (**C**–**E**) Semiquantitative histopathological scores (scale 1–4) for lung inflammation, necrosis, and fibrosis, respectively. (**F**) Representative photomicrographs of H&E-stained lung sections (10× and 40× magnification. Ex vivo cytokine profile (IL-2, IL-4, IL-6, TNF-α, IL-17, IL-10, and IFN-γ) in lung (**G**) and spleen (**H**) culture supernatants (24 h, no additional stimulation). Data are presented as heatmaps with the mean indicated in the boxes. Statistical Analysis: One-way ANOVA followed by post hoc Tukey multiple-comparisons test. Asterisks indicate significance relative to the control group (* *p* ≤ 0.05; ** *p* ≤ 0.01; *** *p* ≤ 0.001); # indicate significance relative to the BCG group (* *p* ≤ 0.05; ** *p* ≤ 0.01; *** *p* ≤ 0.001) brackets indicate specific comparisons between vaccine groups. The heatmap shows cytokine levels as Z-scores relative to saline; Red indicates values above and blue below the saline mean; numbers in each cell indicate the group median Z-score.

**Figure 2:**
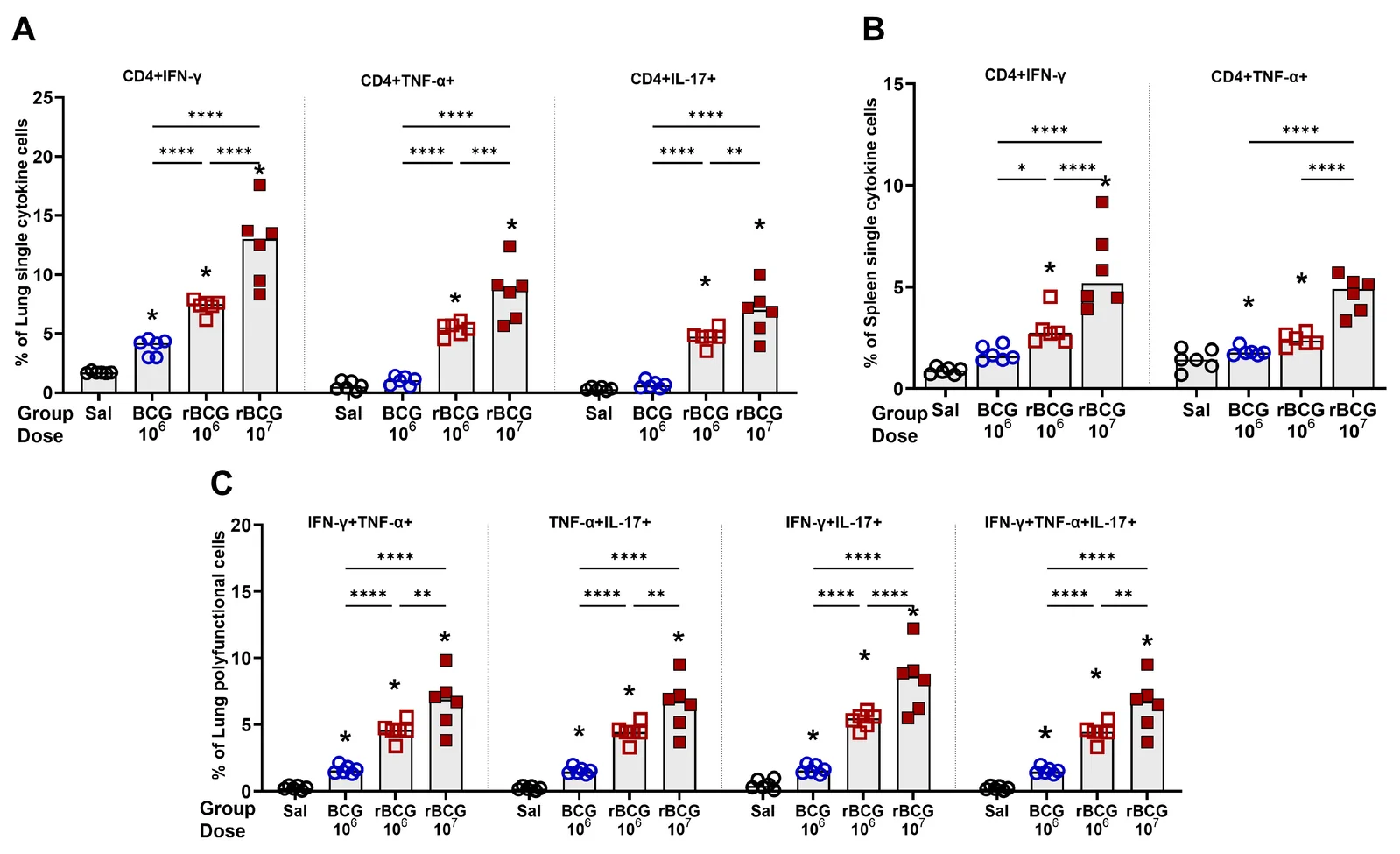
High-dose immunization with rBCG-LTAK63 enhances Th1/Th17 responses in mouse lungs. Mice were immunized subcutaneously with either BCG (10^6^ CFU) or rBCG-LTAK63 (10^6^ or 10^7^ CFU) (*n* = 6 per group). Immune responses were evaluated 90 days post-immunization. Lung and spleen samples were collected, and cellular suspensions were restimulated with CFP to assess CD4^+^ effector T-cell subsets. (**A**) Lung and (**B**) spleen cell subsets following rBCG-LTAK63 immunization: CD4^+^IFN-γ^+^, CD4^+^TNF-α^+^ and CD4^+^IL-17^+^ cells; (**C**) polyfunctional CD4^+^ T-cells in the lungs. Data are displayed as bar plots (representing the mean value) with individual animals’ data overlaid. Significance was determined as *p* ≤ 0.05 relative to the control group (saline) (one-way ANOVA, *n* = 6). Asterisks (*) indicate statistically significant differences between groups (* *p* ≤ 0.05, ** *p* ≤ 0.01, *** *p* ≤ 0.001 and **** *p* ≤ 0.0001). The data shown are representative of two independent experiments.

**Figure 3:**
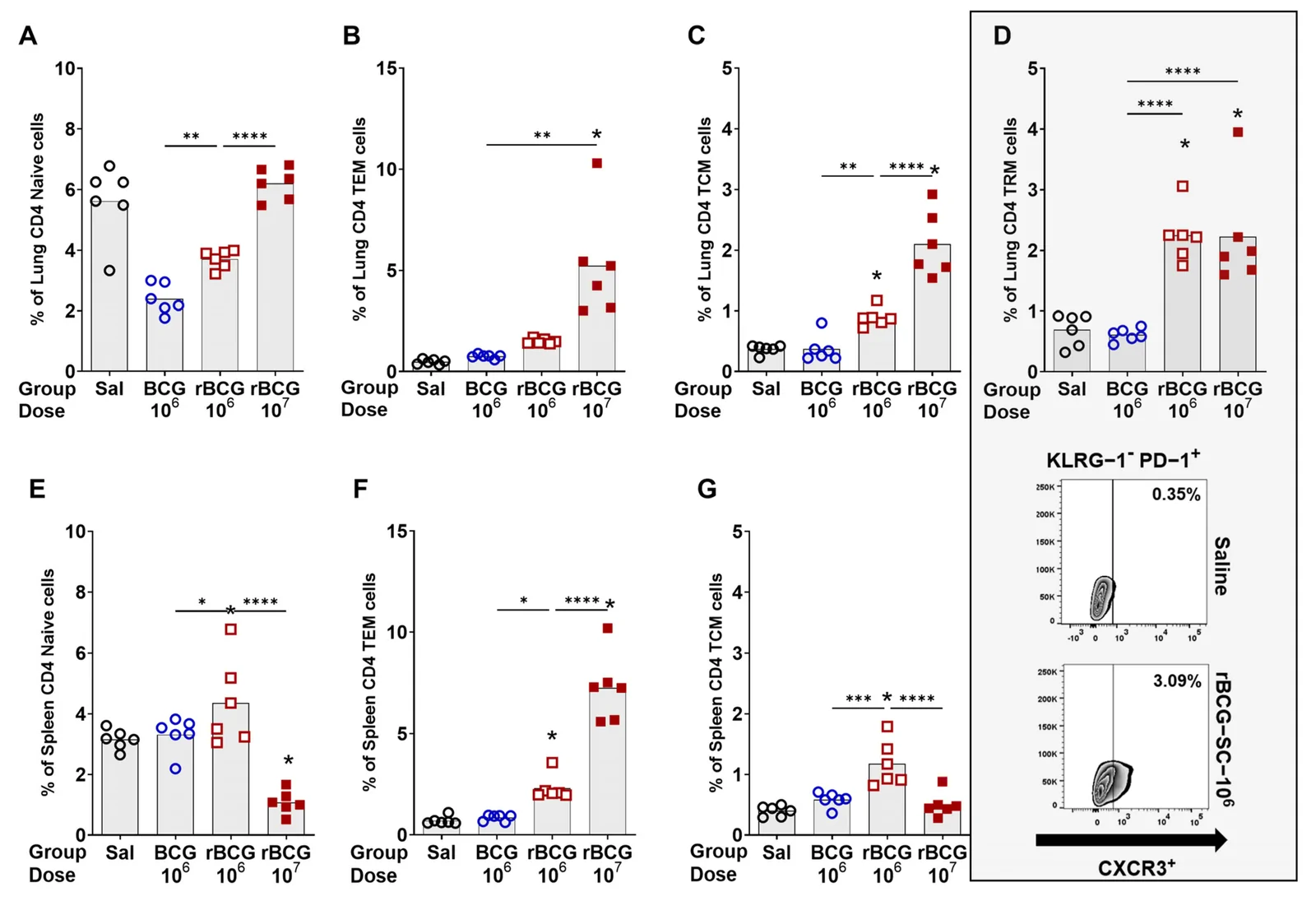
High-dose subcutaneous rBCG-LTAK63 modulates CD4+ naïve and memory T-cell subsets in lung and spleen at day 90 post-immunization. BALB/c mice (*n* = 6 per group) were immunized SC with BCG (1 × 10^6^ CFU) or rBCG-LTAK63 (1 × 10^6^ or 1 × 10^7^ CFU); controls received saline. At day 90, single-cell suspensions from lungs (**A**–**D**) and spleen (**E**–**G**) were re-stimulated ex vivo with CFP and analyzed for CD4^+^ T-cell subsets: naïve ((**A**,**E**); CD3^+^CD4^+^CD44^−^CD62L^+^), central memory—TCM, ((**B**,**F**) CD3^+^CD4^+^CD44^+^CD62L^+^), effector memory—TEM, ((**C**,**G**); CD3^+^CD4^+^CD44^+^CD62L^−^), and lung tissue-resident memory—TRM ((**D**); CD3^+^CD4^+^PD-1^+^KLRG1^−^CXCR3^+^). Data are displayed in bar plots (mean value) with individual animals overlaid. Statistics: one-way ANOVA was performed independently for each panel across groups, followed by post hoc multiple-comparison testing with adjustment for multiplicity (see Section 2: Methods). Two-sided α = 0.05. Asterisks denote adjusted *p*-values for the indicated pairwise comparisons versus saline and/or BCG controls, while brackets indicate the specific contrasts tested (* *p* ≤ 0.05, ** *p* ≤ 0.01, *** *p* ≤ 0.001, **** *p* ≤ 0.0001).

**Figure 4:**
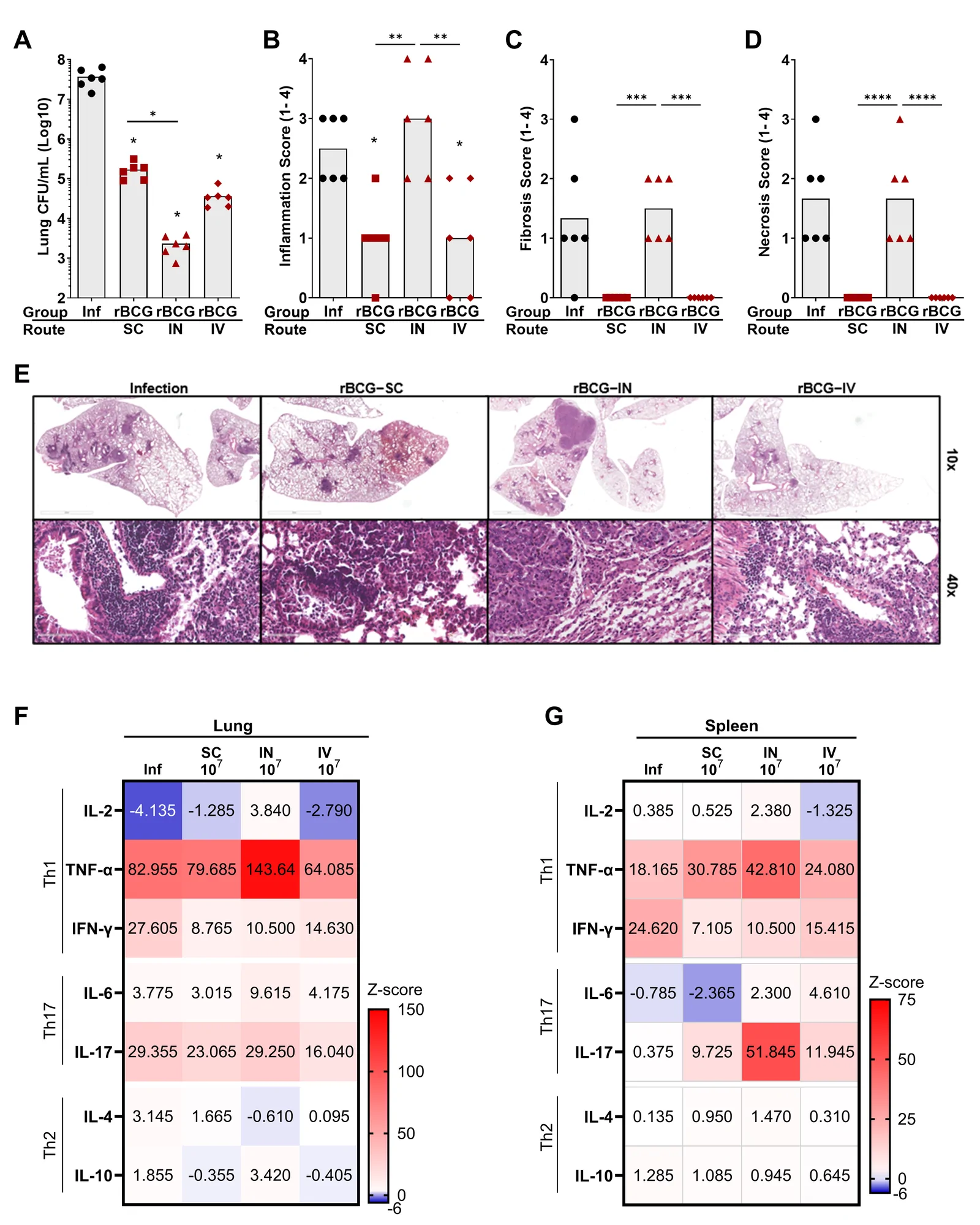
Route of administration dictates protective-versus-pathogenic imprinting by high-dose rBCG-LTAK63. rBCG-LTAK63 was administered by subcutaneous (SC), intranasal (IN), or intravenous (IV) routes (all at 1 × 10^7^ CFU). Ninety days after immunization, animals were intranasally challenged with 500 CFU of *M. tuberculosis*, and protective outcomes were assessed 30 days post-challenge. Panels: (**A**) pulmonary bacterial burden (log10 CFU/mL); (**B**) lung inflammation score; (**C**) lung fibrosis score; (**D**) lung necrosis score; (**E**) representative lung histology (H&E; 10× and 40×) for each group; (**F**) lung cytokine heatmap (IL-2, IL-4, IL-6, TNF-α, IL-17, IL-10, IFN-γ; values shown as Z-scores); (**G**) spleen cytokine heatmap (same analytes; Z-scores). Data in (**A**–**D**) are displayed as bar plots (mean value of data) with individual animals overlaid. One-way ANOVA across groups followed by multiplicity-adjusted post hoc pairwise comparisons. Asterisks above brackets denote *p*-values for the indicated differences (* *p* ≤ 0.05, ** *p* ≤ 0.01, *** *p* ≤ 0.001, **** *p* ≤ 0.0001). The heatmap shows cytokine levels as Z-scores relative to saline; Red indicates values above and blue below the saline; numbers in each cell indicate the group median Z-score.

**Figure 5:**
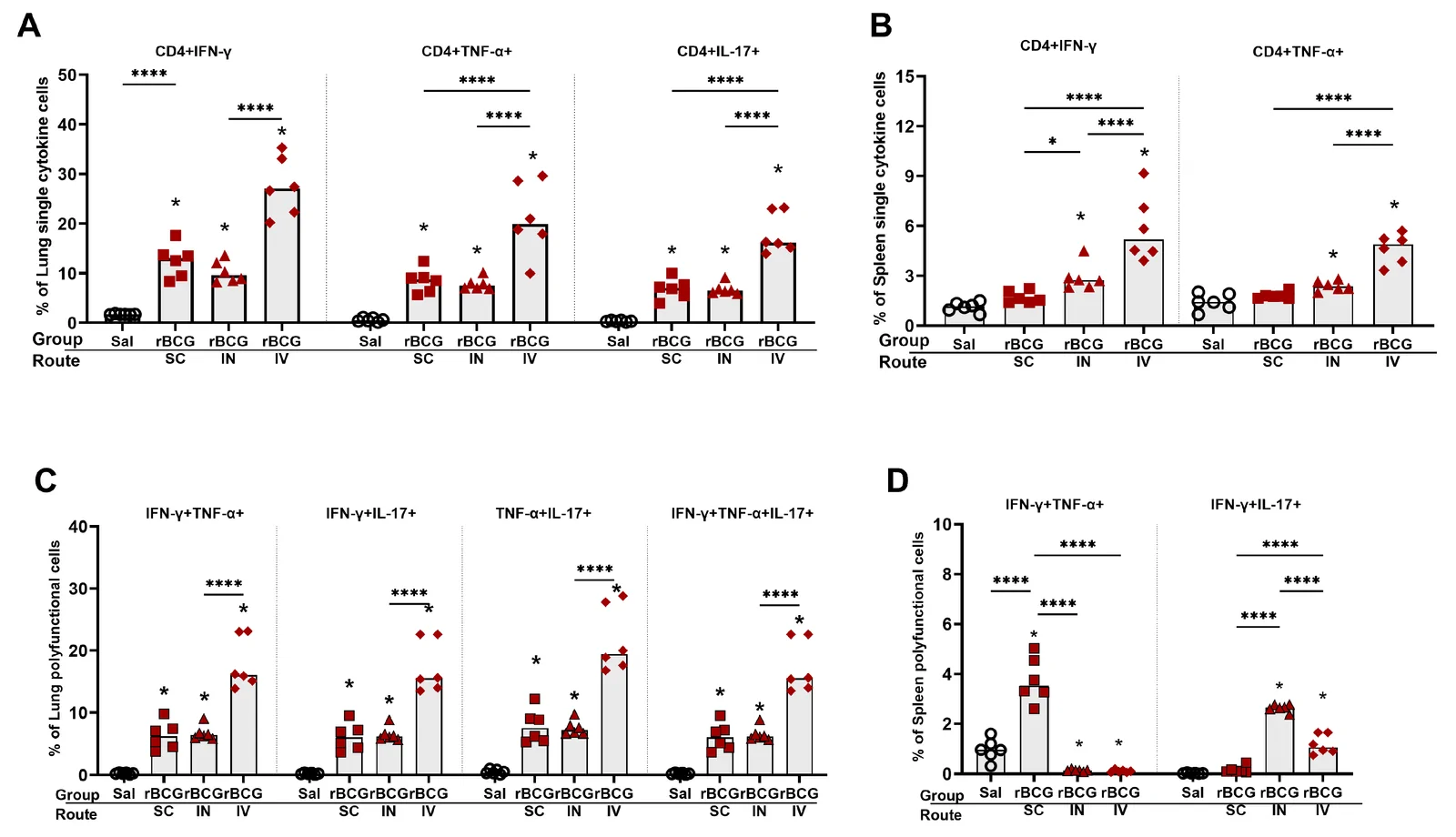
High-dose rBCG-LTAK63 elicits route-dependent CD4+ Th1/Th17 and polyfunctional responses in the lungs and spleen. Animals received rBCG-LTAK63 at a fixed high dose (1 × 10^7^ CFU) via subcutaneous (SC), intranasal (IN), or intravenous (IV) routes. Panel (**A**) shows lung frequency of CD3^+^CD4^+^ IFN-γ^+^, TNF-α^+^ and IL-17^+^ cells, while (**B**) shows the spleen frequency of CD3^+^CD4^+^ IFN-γ^+^ and TNF-α^+^. Panel (**C**) displays the distribution of polyfunctional subsets IFN-γ^+^TNF-α^+^, IFN-γ^+^IL-17^+^, TNF-α^+^IL-17^+^ and IFN-γ^+^TNF-α^+^IL-17^+^ in the lungs, while panel (**D**) displays the distribution of polyfunctional subsets IFN-γ^+^TNF-α^+^ and IFN-γ^+^IL-17^+^. Data are displayed as bar plots with individual animals overlaid, with the top line of the bar representing the mean value of data. One-way ANOVA across groups followed by multiplicity-adjusted post hoc pairwise comparisons were employed. Asterisks above brackets denote *p*-values for the indicated differences (* *p* ≤ 0.05 and **** *p* ≤ 0.0001). The figure is representative of two independent experiments.

**Figure 6:**
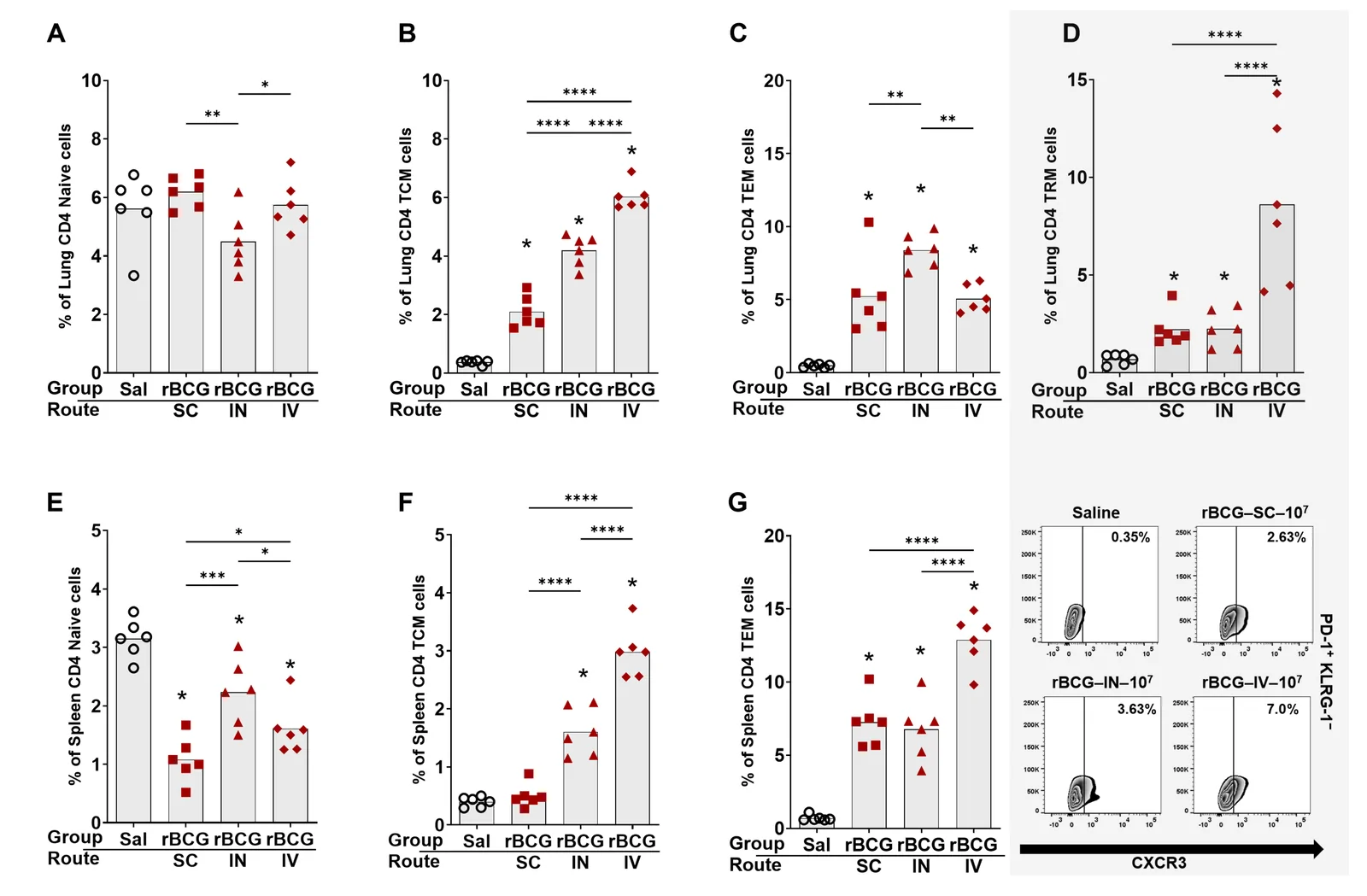
High-dose rBCG-LTAK63 imprints route-dependent CD4^+^ memory profiles in lung and spleen. At a fixed high dose (1 × 10^7^ CFU), rBCG-LTAK63 was administered by subcutaneous (SC), intranasal (IN), or intravenous (IV) routes, and CD4^+^ T-cell memory phenotypes were quantified by flow cytometry. Lung panels (**A**–**D**) show the percentage of CD3+CD4+ cells that are naïve ((**A**); CD44^−^CD62L^+^), central memory ((**B**); CD44^+^CD62L^+^), effector memory ((**C**); CD44^+^CD62L^−^), and tissue-resident memory ((**D**); representative phenotype PD-1^+^KLRG-1^−^CXCR3^+^; gating details in Section 2: Methods). Spleen panels (**E**–**G**) show naïve (**E**), central memory (**F**), and effector memory (**G**) using the same markers. Data are displayed as bar plot plots with individual animals overlaid, with the top line of the bar representing the mean value of data. One-way ANOVA across groups followed by multiplicity-adjusted post hoc pairwise comparisons. Asterisks above brackets denote *p*-values for the indicated contrasts (* *p* ≤ 0.05, ** *p* ≤ 0.01, *** *p* ≤ 0.001, **** *p* ≤ 0.0001). The figure is representative of two independent experiments.

## Data Availability

The raw data supporting the conclusions of this article will be made available by the authors on request.

## Supplementary Materials

The following supporting information can be downloaded at: https://www.mdpi.com/article/10.3390/vaccines14100845/s1. Figure S1: Gating strategy used to identify cytokine-producing CD4^+^ T cells; Figure S2: Representative flow cytometry plot showing lung IFN-γ and TNF-α producing CD4^+^ T cells in the rBCG-IV-10^7^ group compared to the control groups (Saline and rBCG-SC-10^6^); Figure S3: Splenic cytokine producing T cell analysis; Figure S4: Analysis of central and effector memory T cell population; Figure S5: Analysis of tissue resident memory T cell population. The CD3^+^CD4^+^ effector T-cell population was characterized according to the production of IFN-γ, TNF-α, and/or IL-17. Subsequently, the polyfunctional frequency was assessed using a Boolean “and” gate for double or triple-positive cells. The memory helper T-cell population was categorized into subsets: naïve (CD3^+^CD4^+^CD44^−^CD62L^+^), central memory (TCM, CD3^+^CD4^+^CD44^+^CD62L^+^), effector memory (TEM, CD3^+^CD4^+^CD44^+^CD62L^−^), or tissue-resident memory (TRM, CD3^+^CD4^+^PD-1^+^KLRG1^−^ CXCR3^+^) cells. The gating strategies for all subgroups of memory T-cells are shown in Supplementary Figure S1 present the gating strategy employed to identify cytokine-producing CD4^+^; T-cell subsets. Representative flow cytometry plots depicting Th1 and Th17 cytokine frequencies in lung and spleen tissues are shown in Supplementary Figures S2 and S3, respectively, illustrating lung and spleen differences in T helper cell Th1 and Th17 populations. Figure S3B depicts the splenic polyfunctional evaluation. Supplementary Figure S4 displays the gating strategy and representative plots for identifying CD4^+^; T-cell memory phenotypes, including naïve (CD62L^+^; CD44^−^;), central memory (TCM; CD62L^+^CD44^+^), and effector memory (TEM; CD62L^−^CD44^+^) populations, evaluated in animal the spleen and lungs. Supplementary Figure S5 illustrates the identification of tissue-resident memory (TRM) CD4^+^; T-cells, defined by the phenotype PD-1^+^KLRG-1^−^CXCR3^+^, reflecting locally retained T-cell responses within pulmonary tissue.
