# Supplementary material for: Dose and Route of rBCG-LTAK63 Immunization Determine Protective Efficacy and Pathological Outcome in Tuberculosis
Source: Vaccines (Basel). 2026 Sep 24;14(10):845. doi: 10.3390/vaccines14100845 (PMC13709460; doi:10.3390/vaccines14100845)
Supplement: Supplementary file 1 [file vaccines-14-00845-s001.zip › vaccines-4535603-supplementary.pdf]

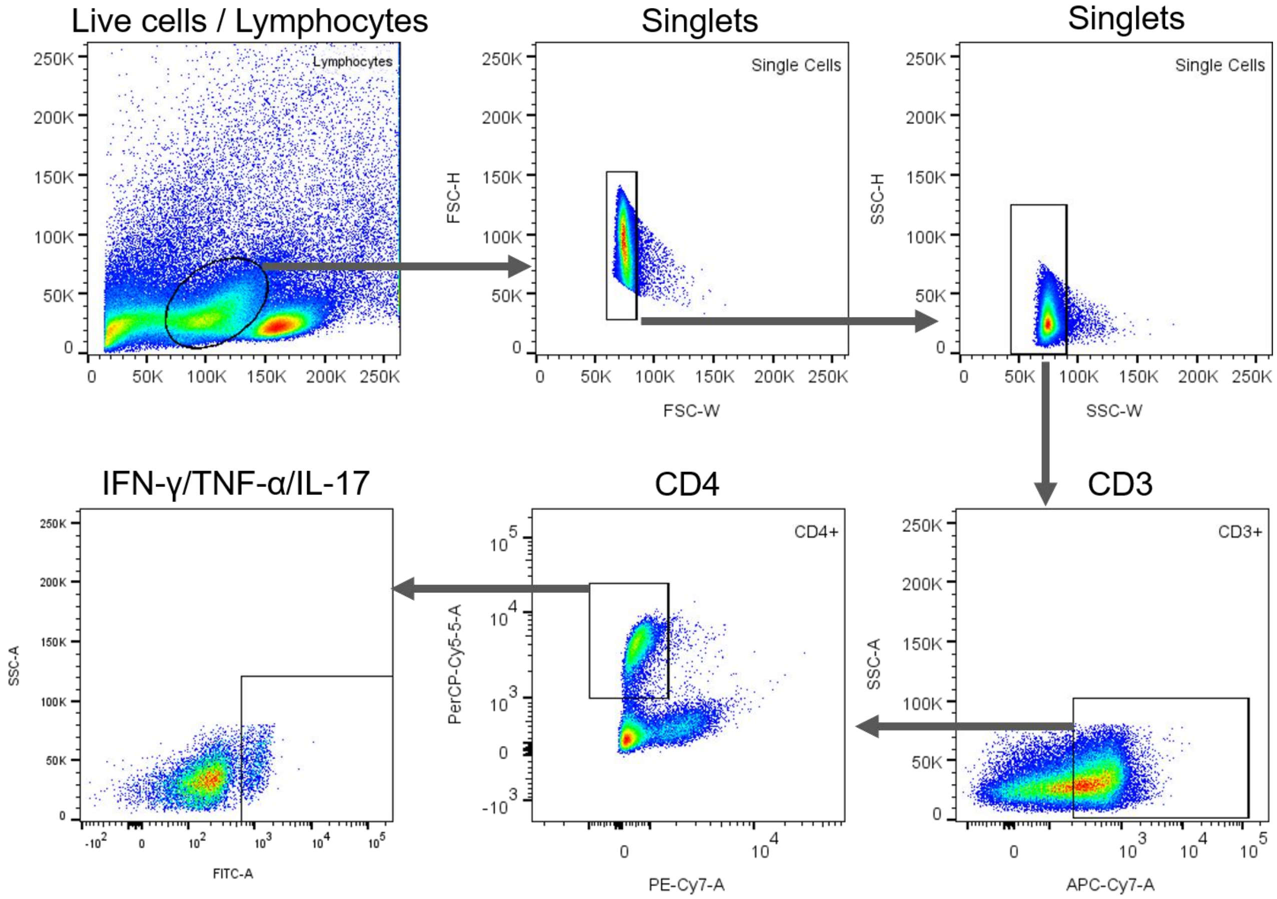

Supplementary Figure S1. Gating strategy used to identify cytokine-producing CD4+ T cells. Initial gates were applied to select live lymphocytes, followed by sequential singlet discrimination using FSC-H × FSC-W and SSC-H × SSC-W. CD3+ T cells were then gated, followed by CD4+ cells, and the frequency of IFN-γ and TNF-α expressing cells was assessed within this subset. All gating steps were performed after the exclusion of dead cells.

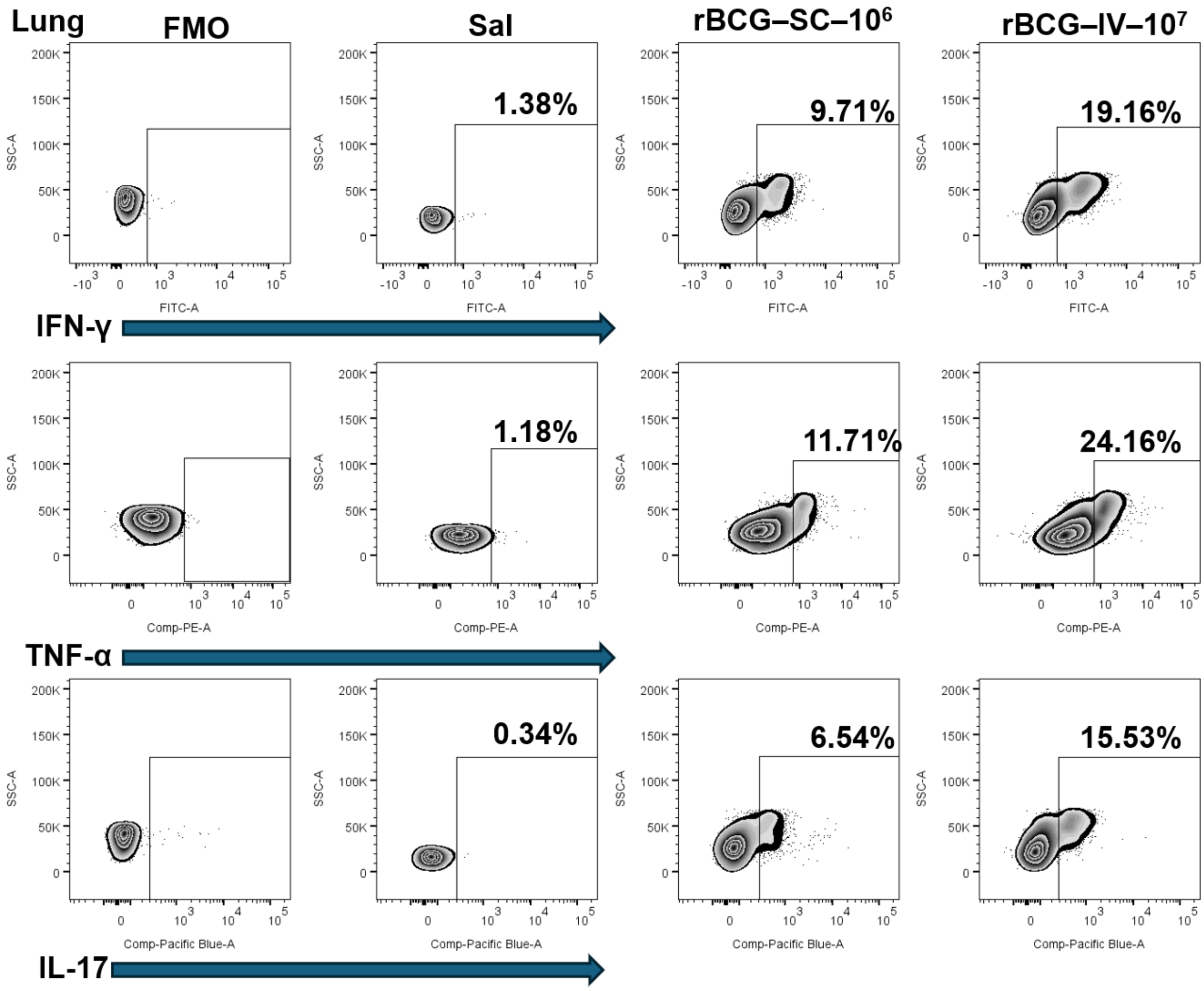

Supplementary Figure S2. Representative flow cytometry plot showing lung IFN-γ and TNF-α producing $CD4^+$ T cells in the rBCG-IV-10^7group compared to the control groups (Saline and rBCG-SC-10^6).The plot corresponds to the group with the most distinct cytokine expression profile (rBCG-IV-10^7), as the other groups showed frequencies similar to rBCG-SC-10^6. Percentages indicate the frequency of cytokine-producing cells relative to the grandparent population (lymphocyte gate). Intracellular cytokine gating was performed based on the FMO (Fluorescence Minus One) strategy.

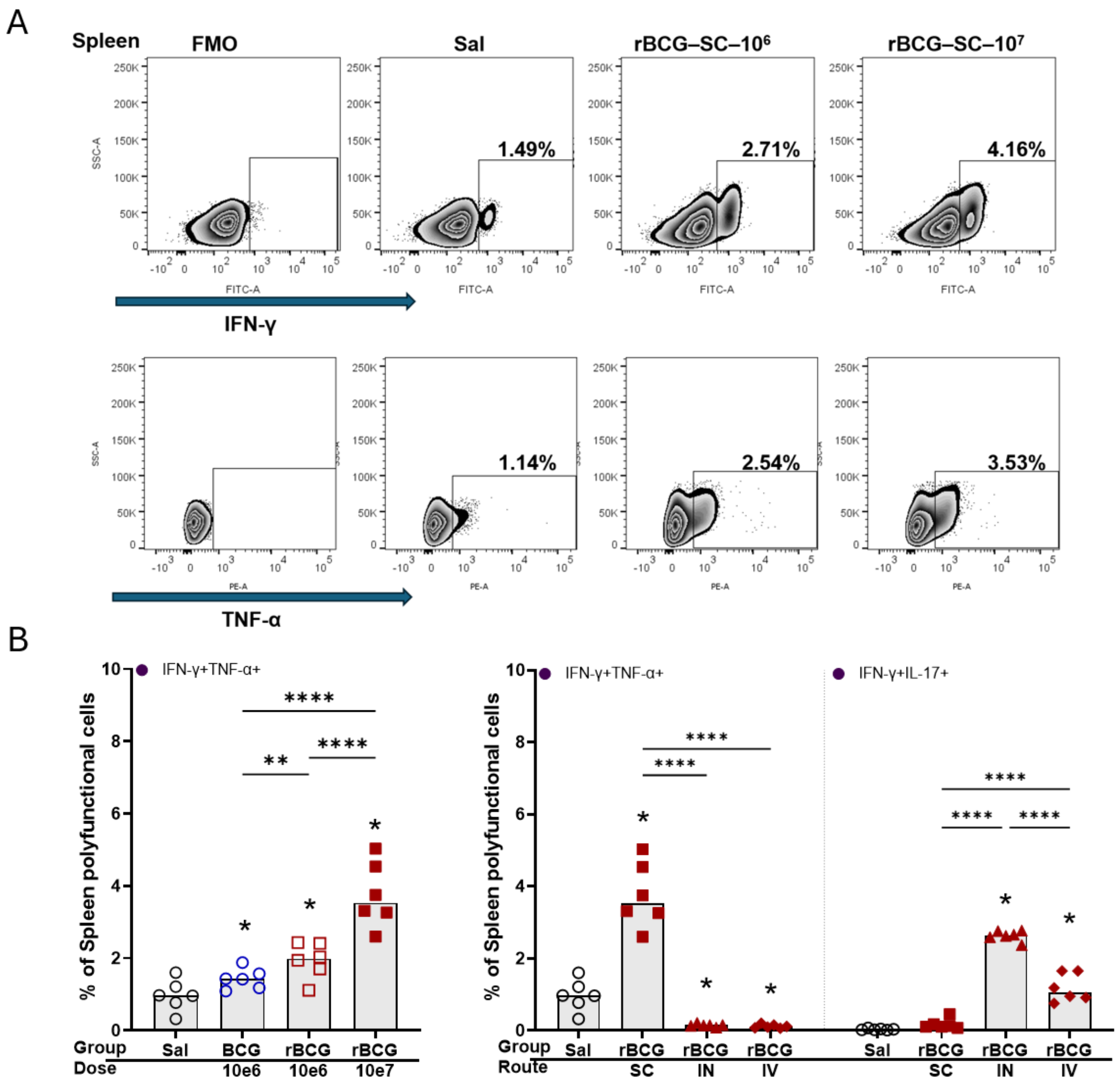

Supplementary Figure S3. Splenic cytokine producing T cell analysis. (A) Representative flow cytometry plot showing splenic IFN-γ and TNF-α producing CD4$^+$ T cells in the rBCG-SC-10^7 group compared to the control groups (Saline and rBCG-SC-10^6). The plot corresponds to the group with the most distinct cytokine expression profile (rBCG-SC-10^7), as the other groups showed frequencies similar to rBCG-SC-10^6. Percentages indicate the frequency of cytokine-producing cells relative to the grandparent population (lymphotes cells). (B) Frequency of polyfunctional cells in the spleen. Data are displayed as violin plots with individual animals overlaid; pie charts summa-rize the proportional composition of dual/triple cytokine positive subsets per route. One-way ANOVA across groups followed by multiplicity-adjusted post-hoc pairwise comparisons. Asterisks above brackets denote p-values for the indicated contrasts (* p ≤ [illegible], ** p ≤ [illegible], *** p ≤ [illegible]). The figure is representative of two independent ex-periments.

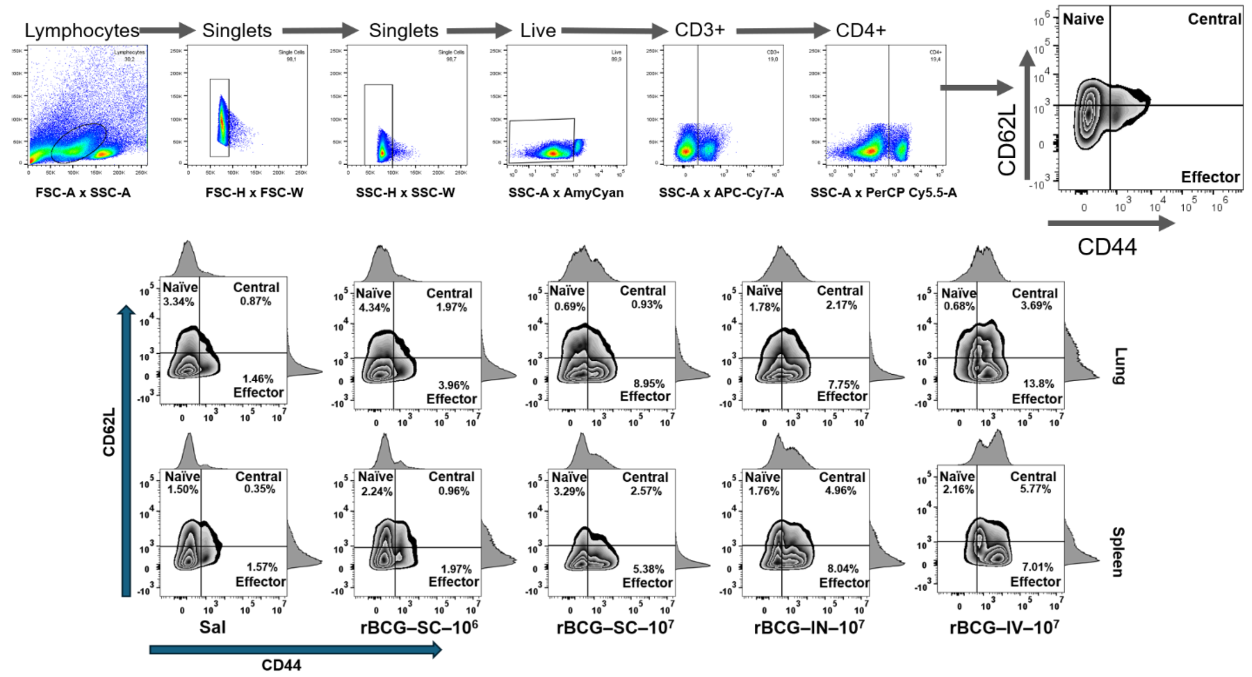

Supplementary Figure S4. Analysis of central and effector memory T cell population. Flow cytometry gating strategy and representative plots showing the distribution of splenic naïve ($CD62L^+CD44^-$), central memory ($CD62L^+CD44^+$), and effector memory ($CD62L^-CD44^+$) $CD4^+$ T cells in the high dose and different route of immunization groups compared to control groups (Saline and rBCG-SC-10^6). The figure upper panel represents the gating strategy used to identify live lymphocytes, followed by singlet discrimination (FSC-H × FSC-W and SSC-H × SSC-W), live cells, then gating of CD3+ cells followed by $CD4^+$ T cells. Memory subsets were defined within the $CD4^+$ population based on CD62L and CD44 expression. The representative plot (lower panel) corresponds to each studied group, which showed the most distinct distribution of memory subsets. Percentages indicate the frequency of each subset relative to the grandparent population (live lymphocytes). Grey histograms flanking each plot show the marginal distributions (one-dimensional projections) of the $CD4^+$ events displayed in the corresponding contour plot: CD44 on top and CD62L on the right.

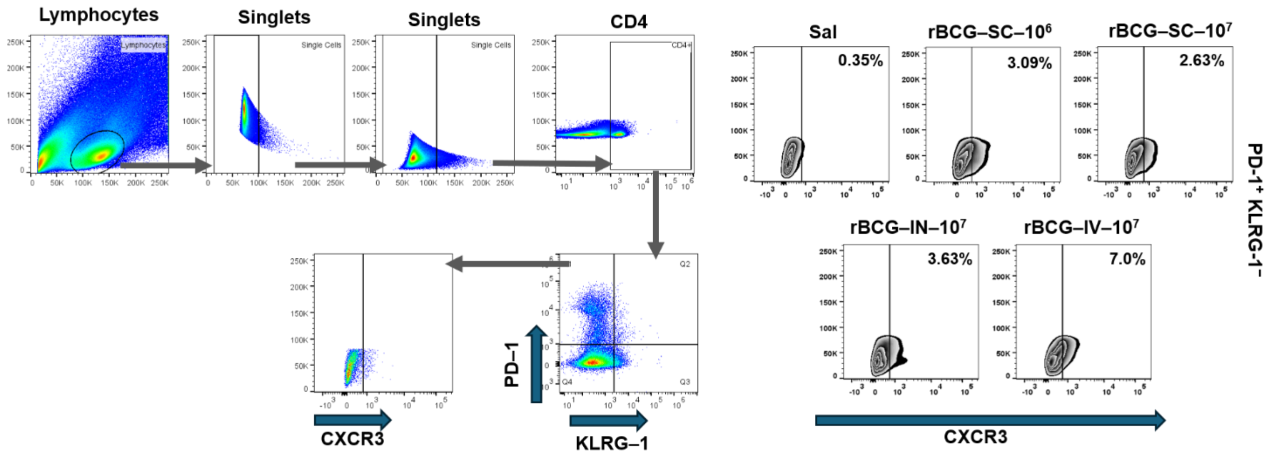

Supplementary Figure S5. Analysis of tissue resident memory T cell population. Flow cytometry gating strategy and representative plots showing the identification of splenic tissue-resident memory $CD4^+$ T cells (TRM), defined as $PD\text{-}1^+$ $KLRG\text{-}1^-$ $CXCR3^+$. The left panel with the gating strategy, which includes the selection of live lymphocytes, singlet discrimination (FSC-H × FSC-W and SSC-H × SSC-W), and gating on $CD4^+$ T cells. Within this population, TRM cells were identified based on co-expression of PD-1 and CXCR3 in the absence of KLRG-1 expression. The representative plot corresponds of all studied groups, which exhibited distinct TRM phenotype. Percentages indicate the frequency of TRM cells relative to the grandparent population (live lymphocytes). All gattings were performed after CD3+ Live cell gatting selection.
